# Naïve Bayesian Classifier and Genetic Risk Score for Genetic Risk Prediction of a Categorical Trait: Not so Different after all!

**DOI:** 10.3389/fgene.2012.00026

**Published:** 2012-02-29

**Authors:** Paola Sebastiani, Nadia Solovieff, Jenny X. Sun

**Affiliations:** ^1^Department of Biostatistics, Boston University School of Public Health Boston, MA, USA; ^2^Psychiatric and Neurodevelopmental Genetics Unit, Center for Human Genetics Research, Massachusetts General Hospital Boston, MA, USA; ^3^Stanley Center for Psychiatric Research, The Broad Institute of MIT and Harvard Cambridge, MA, USA; ^4^Department of Psychiatry, Harvard Medical School Boston, MA, USA

**Keywords:** genetic risk prediction, genetic score, Naïve Bayes classifier, classification score, classification rule

## Abstract

One of the most popular modeling approaches to genetic risk prediction is to use a summary of risk alleles in the form of an unweighted or a weighted genetic risk score, with weights that relate to the odds for the phenotype in carriers of the individual alleles. Recent contributions have proposed the use of Bayesian classification rules using Naïve Bayes classifiers. We examine the relation between the two approaches for genetic risk prediction and show that the methods are mathematically related. In addition, we study the properties of the two approaches and describe how they can be generalized to include various models of inheritance.

## Introduction

Several statistical methods have been proposed to capture the complex genetic bases of common diseases. These approaches include standard regression models in which the contribution of several genetic variants is summarized by a genetic risk score (GRS; Meigs et al., 2008; Purcell et al., 2009; Paynter et al., 2010), multivariate regression models and “machine learning type” approaches such as support vector machines (Wei et al., 2009; Wu et al., 2011), Naïve Bayes classifiers (NBC; Okser et al., 2010), classification and regression trees, random forests (Bureau et al., 2005; McKinney et al., 2006), rule induction (Sebastiani and Perls, 2010; Stengard et al., 2010), multifactor dimensionality reduction (Moore et al., 2006), and Bayesian networks (Rodin and Boerwinkle, 2005; Sebastiani et al., 2005; Jiang et al., 2011; Kang et al., 2011a). NBCs use a simple but surprisingly effective Bayesian rule that classifies a subject at risk of a trait if the posterior probability of the trait, given the individual’s genetic profile, is maximal (Hand, 2009). The classification rule can be built using a large number of genetic variants, such as single nucleotide polymorphisms (SNPs), by assuming that the SNPs are conditionally independent given the trait (Sebastiani et al., 2012). This hypothesis is often mistaken for “marginal independence” but marginal and conditional independence have no relation (Whittaker, 1990).

In this manuscript we show that there is a mathematical link between NBCs and logistic regression models that use a GRS to summarize the contribution of many SNPs to the susceptibility to a genetic disease. The link between these two approaches also highlights their limitations. We discuss how the directed graphical model underlying a NBC can be extended to include interactions between genes and/or environmental risk factors by maintaining the computations scalable to genome-wide genotype data and even whole genome sequence data.

## Methods and Results

We describe two approaches – logistic regression and Bayesian classifier – to define a classification score and a rule to be used for genetic risk prediction of a dichotomous trait denoted as *T* or “not *T*.” The classification score for genetic risk prediction is a function that maps a set of SNPs Σ = {*S*_1_, …, *S_k_*} into real numbers. The classification rule links the output of the score function to the events *T* or “not *T*.” Formally, with S denoting the space of SNPs and *R* the real numbers:


$$\begin{array}{c} \text{Classification score: Sc}\left(\Sigma \right):S\to R\\ \text{Classification rule: Sc(}\Sigma \text{) }>\;\tau \Rightarrow \text{Classify as }T \end{array}$$

### Logistic regression with a genetic risk score

A logistic regression model that includes the general effects of *k* biallelic SNPs Σ = {*S*_1_, …, *S_k_*} to model the odds for a dichotomous trait *T* is defined by the logit equation:


$$\log\left(\frac{p\left(T|\Sigma \right)}{1-p\left(T|\Sigma \right)}\right)=\alpha_{0}+\overset{k}{\underset{j=1}{\sum }}\left(\alpha_{1j}X_{j\text{AB}}+\alpha_{2j}X_{j\text{BB}}\right)\;\text{where }X_{j\text{AB}}=\left\{\begin{array}{c} 1\text{ if }S_{\text{j}}\text{ genotype}=\text{AB}\;\\ \text{0 otherwise}\;\\ \; \end{array}\right.\text{ and }X_{j\text{BB}}=\left\{\begin{array}{c} 1\text{ if }S_{\text{j}}\text{ genotype}=\text{BB}\;\\ \text{0 otherwise}\;\\ \; \end{array}\right.$$

We assume that the alleles of the SNPs are ordered in lexicographical order (A < C < G < T), and A represents the first allele and B the second allele regardless of their frequency. The logit equation is the classification score that can be used to define a classification rule based on a threshold τ:


$$\text{Classification score: Sc}\left(\Sigma \right)=\log\left(\frac{p\left(T|\Sigma \right)}{1-p\left(T|\Sigma \right)}\right)=\alpha_{0}\;+\sum_{j}\left({\alpha_{1j}}^{X_{j\text{AB}}}+{\alpha_{2j}}^{X_{j\text{BB}}}\right)\text{Classification rule: Sc(}\Sigma \text{) }>\;\tau \Rightarrow \text{Classify as }T$$

and τ can be determined to optimize sensitivity and specificity by receiver operating characteristic (ROC) curve analysis.

The coefficients of the logistic score are typically estimated by maximum likelihood (McCullagh and Nelder, 1989), or Bayesian methods using large sample approximations or Gibbs sampling (Balding, 2006). By definition, the intercept α_0_ represents the log-odds for the trait *T* for the referent group with all SNPs genotypes equal to AA, while each parameter α_1*j*_ represents the log-odds ratio for the trait *T* between the AB genotype and the AA genotype of the *j*th SNP, and each parameter α_2*j*_ represents the log-odds ratio for *T* between the BB and AA genotype of the *j*th SNP, assuming the other SNP genotypes fixed. When α_2*j*_ = 2α_1*j*_ for all *j* = 1, …, *k*, then the logistic regression encodes the additive effects of the SNPs, and each parameter α_1*j*_ represents the log-odds ratio for *T* for each additional copy of the B allele relative to the referent genotype AA.

It is well known that when the data are from a case–control study design, the intercept does not provide the correct estimate of the odds for *T* in the populations and several corrections have been proposed to limit this problem (Jewell, 2003). Bias of the intercept term is not a problem when the logistic regression model is meant to be used for classification because different intercepts will simply shift the logistic function and classification scores that differ only by the intercept term lead to equivalent classification rules. We state this property formally because it will be used further.

#### Property 1: Irrelevance of the intercept term of a logistic regression model for classification

Let Sc_1_(Σ) and Sc_2_(Σ) be two classification scores defined as:


$$\text{S}{\text{c}}_{1}\left(\Sigma \right)=\log\left(\frac{p\left(T|\Sigma \right)}{1-p\left(T|\Sigma \right)}\right)=\alpha_{0}+\sum_{j}\left(\alpha_{1j}X_{j\text{AB}}+\alpha_{2j}X_{j\text{BB}}\right)\text{S}{\text{c}}_{\text{2}}\left(\Sigma \right)=\log\left(\frac{p\left(T|\Sigma \right)}{1-p\left(T|\Sigma \right)}\right)=\beta_{0}+\sum_{j}\left(\alpha_{1j}X_{j\text{AB}}+\alpha_{2j}X_{j\text{BB}}\right)$$

The two classification scores can be used to define equivalent classification rules by using the relation:

$$\begin{array}{rcll} & \text{“if S}{\text{c}}_{\text{1}}\left(\Sigma \right)>\tau \Rightarrow \text{classify as T”}\text{ if and only if} & & \\ & \text{“if S}{\text{c}}_{\text{2}}\left(\Sigma \right)>\tau +\beta_{0}-\alpha_{0}\Rightarrow \text{classify as }\text{T”}\;□ & & \end{array}$$

We note however that the correct estimate of the intercept term is necessary to be able to interpret the prediction from the logistic model in terms of prevalence of the trait in the population.

One of the limitations of multivariate logistic regression is that the number of covariates is bounded above by the sample size. It is expected that many common genetic complex traits may be determined by hundreds of genetic variants (Kraft and Hunter, 2009), so that the sample size needed to build reliable logistic regression models for risk prediction can be prohibitively large.

A naïve but very popular alternative is to collapse the contribution of the *k* SNPs into a GRS to be used in a univariate logistic model. A GRS is typically defined as the weighted sum of the genotypes:


$$\text{GRS}=\text{GRS}\left(\Sigma \right)=\overset{k}{\underset{i=1}{\sum }}\left(w_{i}X_{i\text{AA}}+v_{i}X_{i\text{AB}}+z_{i}X_{i\text{BB}}\right)\;$$

with weights that can be appropriately chosen. The variables *X_iAB_* and *X_iBB_* are defined as above, and *X_iAA_* = 1 if the *i*th SNP genotype is AA and 0 otherwise. See Table 1 for a summary of three possible weighting schemes. The GRS is then used as risk factor to define a classification score using a univariate logistic regression:

**Table 1:** **Example of choice of weights for the weighted genetic risk score**.

Case	*w_i_*	*v_i_*	*z_i_*	Comments
1	2δ(A = R)	1	2δ(B = R)	R denotes the risk allele and δ(*X* = *Y*) = 1 if *X* = *Y* is true and 0 otherwise
2	0	$V_{i}=\log\frac{\frac{p(T|X_{i}=1)}{1-p(T|X_{i}=1)}}{\frac{p(T|X_{i}=0)}{1-p(T|X_{i}=0)}}$	2*v_i_*	*X_i_* = 0 when the *i*th SNP genotype is AA, and *X_i_* = 1 when the genotype is AB. This is the standard coding for an additive model
3	0	$\log\frac{\frac{p(T|{\text{S}}_{i}=\text{AB})}{1-p(T|{\text{S}}_{i}=\text{AB})}}{\frac{p(T|{\text{S}}_{i}=\text{AA})}{1-p(T|{\text{S}}_{i}=\text{AA})}}$	$\log\frac{\frac{p(T|{\text{S}}_{i}=\text{BB})}{1-p(T|{\text{S}}_{i}=\text{BB})}}{\frac{p(T|{\text{S}}_{i}=\text{AA})}{1-p(T|{\text{S}}_{i}=\text{AA})}}$	The two weights represent the log-odds ratio relative to the referent genotype AA. This is the coding for genotypic model.

*Case 1 is known as the “unweighted score” and case 2 is typically referred to as the “weighted genetic risk score.” Case 3 is the most general and flexible but it does not seem to be used*.


$$\text{Sc}\left(\Sigma \right)=\log\left(\frac{p\left(T|\text{GRS}\right)}{1-p\left(T|\text{GRS}\right)}\right)=\gamma_{0}+\gamma_{1}\text{ GRS}$$

##### Case 1

**Figure 1:**
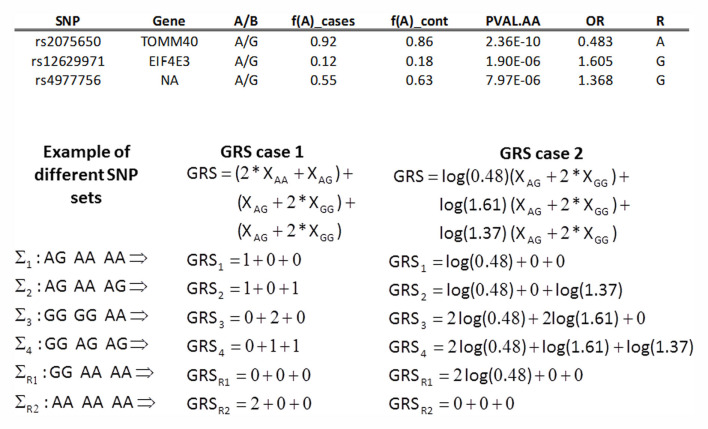
**Example of GRS (case 1 and case 2 in Table 1) based on three SNPs associated with exceptional longevity**. The table on top reports the A/B alleles for the three SNPs, the frequencies of A allele in cases and controls, and the *p*-value for the additive model (Column PVAL.AA) and the odds ratio (OR) for exceptional longevity in carriers of the B allele. The two bottom panels show the calculations of the GRS with weights as in case 1 (left), and case 2 (right). Note that the GRS on the left is only a function of the different number of risk alleles regardless of their individual genetic effects, so the genetic profiles Σ_2_, Σ_3_, and Σ_4_ have the same score while the case 2 GRS assigns different weights to non-referent genotypes and the scores are different. The profile Σ_R1_ denotes the referent group in case 1, while Σ_R2_ denotes the referent group in case 2. The data for this example are taken from Sebastiani et al. (2012).

Although this is often referred to as the “unweighted genetic score,” the heterozygote genotype is always assigned a weight 1, while the homozygous genotype for the risk allele is assigned weight 2 and the other genotype is assigned weight 0. By adopting this weighting scheme, we are simply counting the number of risk alleles each subject carries. The risk allele of each SNP is determined by a “one-SNP-at-a-time” association analysis, typically under an additive genetic model. Using the same notation and lexicographical order of the SNPs that we used earlier, the risk allele of each SNP will be the A allele if the regression coefficient α*_i_* of the logistic regression model


$$\log\left(\frac{p\left(T|S_{i}\right)}{1-p\left(T|S_{i}\right)}\right)=\alpha_{0i}+\alpha_{i}\left(X_{i\text{AB}}+2X_{i\text{BB}}\right)\;$$

is negative, and the B allele if α*_i_* is positive. In the first case (α*_i_* < 0), each copy of the B allele decreases the odds for *T*, while in the second case (α*_i_* ≥ 0) each copy of the B allele increases the odds for *T*. With this definition, the GRS is only a function of the different number of risk alleles regardless of their individual genetic effects, and two identical GRS values can represent genetic profiles that are substantially different. See Figure 1 for an example.

The slope γ_1_ in the classification score:


(1) $$\text{Sc}\left(\Sigma \right)=\log\left(\frac{p\left(T|\text{GRS}\right)}{1-p\left(T|\text{GRS}\right)}\right)=\gamma_{0}+\gamma_{1}\text{ GRS}$$

measures the association of the GRS with the trait *T* in terms of log-odds ratio for *T* between two GRS that differ by 1, and it is often estimated to test whether the GRS is significantly associated with *T*. However, the value of γ_1_ is irrelevant for classification because two classification scores defined as inEq. 1that differ by the slope will produce equivalent classification rules. This is stated in the next property.

#### Property 2: Irrelevance of the slope of a univariate logistic regression model for classification

Let Sc_1_(Σ) and Sc_2_(Σ) be two classification scores defined as:


$$\text{S}{\text{c}}_{1}\left(\Sigma \right)=\log\left(\frac{p\left(T|\text{GRS}\right)}{1-p\left(T|\text{GRS}\right)}\right)=\gamma_{0}+\gamma_{1}\;\text{GRS}\text{S}{\text{c}}_{2}\left(\Sigma \right)=\log\left(\frac{p\left(T|\text{GRS}\right)}{1-p\left(T|\text{GRS}\right)}\right)=\beta_{0}+\beta_{1}\;\text{GRS}$$

The two classification scores can be used to define equivalent classification rules by using the relation:


$$\begin{array}{rcll} & \text{“S}{\text{c}}_{1}\left(\Sigma \right)>\tau \Rightarrow \text{classify as T”},\text{ if and only if} & & \\ & \text{“S}{\text{c}}_{2}\left(\Sigma \right)>\beta_{0}+\beta_{1}\frac{\tau -\gamma_{0}}{\gamma_{1}}\Rightarrow \text{classify as T”}\;□ & & \end{array}$$

The GRSs labeled 2 and 3 in Table 1 weight SNP alleles in different ways to reflect their individual associations with the trait *T*.

##### Case 2

The GRS can be written as:


$$\text{GRS}=\overset{k}{\underset{i=1}{\sum }}v_{i}\left(X_{i\text{AB}}+2X_{i\text{BB}}\right)\;$$

where each weight *v_i_* is the maximum likelihood estimate of the regression coefficient in the univariate logistic regression:


$$\log\left(\frac{p\left(T|X_{i}\right)}{1-p\left(T|X_{i}\right)}\right)=\alpha_{i0}+v_{i}X_{i};\;X_{i}=\left\{\begin{array}{cc} 1\; & \text{ if }S_{i}=\text{AB}\\ 2\; & \text{ if }S_{i}=\text{BB}\\ \text{ 0}\; & \text{ otherwise}\\ \; \end{array}\right.$$

that measures the association between SNP *S_i_* and the trait *T* with an additive genetic model. Therefore, each weight


$$v_{i}=\log\frac{\frac{p(T|X_{i}=1)}{1-p(T|X_{i}=\text{1})}}{\frac{p(T|X_{i}=\text{0})}{1-p(T|X_{i}=\text{0})}}$$

estimates the log-odds ratio for *T* for each copy of the B allele in an additive genetic model. Note that this formulation of the GRS does not require the specification of the risk allele of the SNPs, and the weighted genetic score will increase by *v_i_* for each copy of the B allele of SNP *S_i_*, if this is a risk allele, and decrease by *v_i_* for each copy of the B allele if this is the protective allele. See the example in Figure 1.

The classification score based on this GRS is computed using the logistic regression inEq. 1, with parameters γ_0_, γ_1_ that can be estimated by maximum likelihood or Bayesian methods. The slope represents the odds ratio (OR) for *T* for a unit change of the GRS. In general, the OR for *T* between two genetic profiles Σ_1_ = {*S*_11_, …, *S*_*k*1_} and Σ_2_ = {*S*_12_, …, *S*_*k*2_} associated with GRS_1_ and GRS_2_ is


$$\begin{array}{l} \log\left(\frac{p(T|{\text{GRS}}_{1})/(1-p(T|{\text{GRS}}_{1})}{p(T|{\text{GRS}}_{2})/(1-p(T|{\text{GRS}}_{2})}\right)\\ \;=\gamma_{1}\sum_{i=1}^{k}\log\left(\frac{p(T|S_{i1})/(1-p(T|S_{i1})}{p(T|S_{i2})/(1-p(T|S_{i2})}\right) \end{array}$$

and this equation shows that the log-odds ratio for *T* between two weighted GRSs is an average of log-odds ratios of the individual genetic effects rescaled by the coefficient γ_1_.

The classification rule


$$\text{if S}{\text{c}}_{1}\left(\Sigma \right)=\log\left(\frac{p\left(T|\text{GRS}\right)}{1-p\left(T|\text{GRS}\right)}\right)>\tau \Rightarrow \text{classify as }T\text{, }$$

based on the score


$$\text{S}{\text{c}}_{1}\left(\Sigma \right)=\log\left(\frac{p\left(T|\text{GRS}\right)}{1-p\left(T|\text{GRS}\right)}\right)=\gamma_{0}+\gamma_{1}\;\text{GRS}$$

is equivalent to:


$$\begin{array}{l} \text{if}\sum_{i=1}^{k}\log\frac{p(T|S_{i})/(1-p(T|S_{i})}{p(T|S_{i}=\text{AA})/(1-p(T|S_{i}=AA)}>\frac{\tau -\gamma_{0}}{\gamma_{1}}\\ \;\Rightarrow \text{ classify as }T \end{array}$$

So the classification rule that uses the weighted GRS in case 2 is essentially based on an average of the individual log-odds ratio for *T* of each SNP genotype relative to the referent genotypes.

##### Case 3

**Figure 2:**
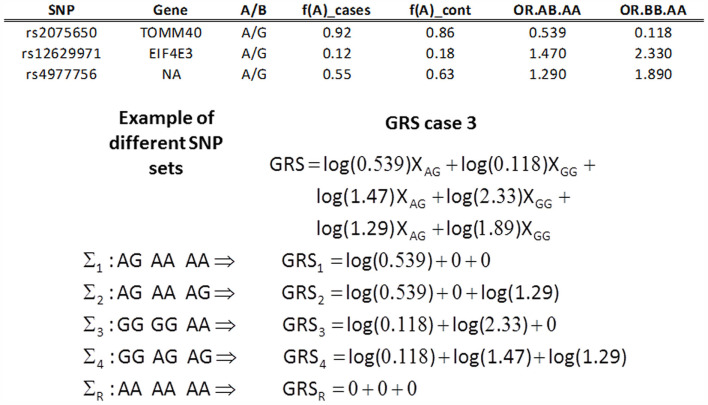
**Example of GRS (case 3 in Table 1)**. The table on top reports the A/B alleles for the three SNPs, the frequencies of A allele in cases and controls, and the odds ratio for exceptional longevity in carriers of the AB allele relative to carriers of the AA allele (OR.AB.AA), and the odds ratio for exceptional longevity in carriers of the BB allele relative to carriers of the AA allele (OR.BB.AA). The bottom panel shows the calculations of the GRS with weights as in case 3. The profile Σ_R_ denotes the referent group.

The GRS is:


$$\text{GRS}=\overset{k}{\underset{i=1}{\sum }}\left(v_{i}X_{i\text{AB}}+z_{i}X_{i\text{BB}}\right)\;$$

where *v_i_* and *z_i_* are the MLE estimate of the regression coefficients of the univariate logistic regression


$$\begin{array}{rcll} & \log\left(\frac{p\left(T|S_{i}\right)}{1-p\left(T|S_{i}\right)}\right)=\alpha_{i0}+v_{i}X_{i\text{AB}}+z_{i}X_{i\text{BB}}; & & \\ & \;\;X_{i\text{AB}}=\left\{\begin{array}{cc} 1\; & \text{if }S_{i}=\text{AB}\\ 0\; & \text{otherwise}\\ \; \end{array}\right.;X_{i\text{BB}}=\left\{\begin{array}{cc} 1\; & \text{if }S_{i}=\text{BB}\\ 0\; & \text{otherwise}\\ \; \end{array}\right. & & \end{array}$$

that measures the genotypic association between SNP *S_i_* and the trait *T*. Therefore


$$v_{i}=\log\frac{\frac{p\left(T|S_{i}=\text{AB}\right)}{1-p\left(T|S_{i}=\text{AB}\right)}}{\frac{p\left(T|S_{i}=\text{AA}\right)}{1-p\left(T|S_{i}=\text{AA}\right)}};\;z_{i}=\log\frac{\frac{p\left(T|S_{i}=\text{BB}\right)}{1-p\left(T|S_{i}=\text{BB}\right)}}{\frac{p\left(T|S_{i}=\text{AA}\right)}{1-p\left(T|S_{i}=\text{AA}\right)}}$$

are the log-odds ratio for *T* between the AB and AA genotypes, and BB and AA genotypes. See Figure 2 for an example. The classification score and classification rule are derived as in case 2 and can be interpreted as average of the log-odds ratios of individual SNPs genotypes. Compared to case 2, the weights based on genotype associations allow for more general model of associations that are not restricted to linear increase of the log-odds for *T*. Note also that when the SNPs included in a GRS (case 2 and 3) are independent, the two scores should be approximately equivalent to multivariate logistic regression with additive (case 2) or genotypic association (case 3). In addition, if the SNPs included in the GRS have similar effects, then the GRS in case 1 and 2 should be approximately equivalent.

### Naïve bayes classifiers

The classification score based on a NBC is the posterior probability of the trait *T* that is calculated using the formula:


$$\text{Sc}(\sum )=p(T|\sum )=\frac{p(T)\prod_{i=1}^{k}p(S_{i}|T)}{\begin{array}{l} p(T)\prod_{i=1}^{k}p(S_{i}|T)\\ \;+(1-p(T))\prod_{i=1}^{k}p(S_{i}|notT) \end{array}}$$

where *p*(*T*) and 1 − *p*(*T*) are the prior probabilities of having the trait *T* or not. The conditional probabilities *p*(*S_i_* | *T*) and *p*(*S_i_* | not *T*) represent the distribution of the *i*th SNP genotype in subjects with and without the trait *T*. They are typically estimated assuming genotypic association (Sebastiani et al., 2012), but they could also be estimated using an additive genetic model. The formula is derived using Bayes’ theorem and assuming that the SNPs are independent, conditionally on *T* (Hand, 2009). The usual Bayesian classification rule is to classify a subject with the most probable outcome


if Sc(Σ)>0.5⇒classify as T.

This rule is based on a 0–1 loss that assigns the same weight to misclassification errors. A general loss function that weights differently sensitivity and specificity would lead to the classification rule:


$$\text{if Sc}\left(\Sigma \right)>\frac{\lambda }{1+\lambda }\Rightarrow \text{classify as }T\text{ for }\lambda >\text{0}$$

that can also be written as:


$$\text{Sc}\left(\Sigma \right)>\frac{\lambda }{1+\lambda }\Leftrightarrow \log\left(\frac{p\left(T|\Sigma \right)}{1-p\left(T|\Sigma \right)}\right)>\log\left(\lambda \right)$$

and simple algebra shows that this is equivalent to:


$$\log\left(\frac{p\left(T|\Sigma \right)}{1-p\left(T|\Sigma \right)}\right)=\log\left(\frac{p\left(T\right)\overset{k}{\underset{i=1}{\prod }}p\left(S_{i}|T\right)}{\left(1-p\left(T\right)\right)\overset{k}{\underset{i=1}{\prod }}p\left(S_{i}|\text{not }T\right)}\right)=\log\left(\frac{\overset{k}{\underset{i=1}{\prod }}p\left(T\right)p\left(S_{i}|T\right)}{\overset{k}{\underset{i=1}{\prod }}\left(1-p\left(T\right)\right)p\left(S_{i}|\text{not }T\right)}\right)=\log\left(\prod_{i=1}^{k}\frac{p\left(T|S_{i}\right)}{1-p\left(T|S_{i}\right)}\right)=\overset{k}{\underset{i=1}{\sum }}\log\left(\frac{p\left(T|S_{i}\right)}{1-p\left(T|S_{i}\right)}\right)>\log\left(\lambda \right)$$

As long as the log-odds ratios are calculated using the same genetic model, this classification rule is equivalent to the classification rule based on the GRS (either case 2 or 3)


$$\begin{array}{rcll} & \text{if }\overset{k}{\underset{i=1}{\sum }}\log\frac{p\left(T|S_{i}\right)∕\left(1-p\left(T|S_{i}\right)\right)}{p\left(T|S_{i}=\text{AA}\right)∕\left(1-p\left(T|S_{i}=\text{AA}\right)\right)}>\frac{\tau -\gamma_{0}}{\gamma_{1}} & & \\ & \;\Rightarrow \text{classify as }T & & \end{array}$$

by setting the threshold


$$\tau =\gamma_{0}-\gamma_{1}\overset{k}{\underset{i=1}{\sum }}\log\left(\frac{p\left(T|S_{i}=\text{AA}\right)}{1-p\left(T|S_{i}=\text{AA}\right)}\right)+\gamma_{1}\log\left(\lambda \right)$$

We state this relation formally.

#### Property 3: Equivalence of classification rules based on the GRS and the NBC

The classification rules based on a logistic model of a GRS(case 2 or 3) and a NBC are equivalent when the same genetic models are used to link individual SNPs to the trait.

The details of the algebraic manipulations are in Section “Appendix.”□

Note that the equivalence between the classification rules based on a NBC and a logistic regression model with a GRS as in case 2 or 3 is a simple consequence of the fact that both models base the prediction on a weighted average of ORs of the individual SNPs. This equivalence is independent of the choice of the prior for *T* because different prior distributions will lead to equivalent classification rules but with different classification thresholds. Also, the equivalence of classification rules based on GRS and NBC implies that when alternative classifiers are compared by the area under the receiving operator curve they must reach the same value. This is shown in the next example.

##### Example

To demonstrate the connection between the NBC and the GRS in case 3, we performed a simple simulation. We simulated a dataset with 3000 cases and 3000 controls, and genotype data from 75 causal SNP and 500,000 null SNPs. For the null SNPs, we randomly selected frequencies of the minor allele (*p*) from a uniform (0.05, 0.5) distribution and genotype frequencies were generated assuming Hardy–Weinberg equilibrium [*p*^2^,2*p*(1 − *p*),(1 − *p*)^2^]. The causal SNPs were simulated with ORs of 1.2, 1.3, 1.4, 1.5, and 1.6 and minor allele frequencies (MAFs) of 0.1, 0.2, 0.3, 0.4, and 0.5. A causal SNP was simulated for each combination of the above ORs and MAFs (25 combinations) under an additive, recessive and dominant mode of inheritance (25 combinations × 3 modes of inheritance = 75 SNPs). The genotype frequencies in controls were generated to follow Hardy–Weinberg equilibrium [*p*^2^,2*p*(1 − *p*),(1 − *p*)^2^]. The genotype frequencies in cases for the additive, recessive, and dominant models were [*p*^2^,2OR*p*(1 − *p*),OR^2^(1 − *p*)^2^], [*p*^2^,2*p*(1 − *p*),OR(1 − *p*)^2^] and [*p*^2^,2OR*p*(1 − *p*),OR(1 − *p*)^2^], respectively. For the cases, the genotype frequencies were divided by the sum of the frequencies so that the frequencies add up to 1. Using the genotype frequencies for each SNP, we simulated a discovery set of 3000 cases and 3000 controls and a replication set with the same sample sizes.

The data in the discovery set were analyzed to generate genetic risk models based on GRS and NBCs in the following way. A Bayesian genome-wide association study was performed on the discovery set and SNPs were ordered according to the posterior probability for the genotypic association to build nested NBCs with increasing number of SNPs as in Sebastiani et al. (2012). To obtain the weights for the three GRSs, we ran two logistic regression models for each SNP, using an additive mode of inheritance and a genotypic mode of inheritance. The results of these analyses were used to detect the risk alleles of SNPs for nested GRS as in case 1; and to estimate the weights of GRS as in cases 2 and 3. Using SNPs ordered by the posterior probability for the genotypic association, we then built three sets of classification models based on logistic regression and the three different GRS, with increasing number of SNPs. The prediction models were tested on the replication set to avoid issues of over-fitting. The simulation described above was repeated five times and the mean AUC across the replicates was used to assess accuracy.

**Figure 3:**
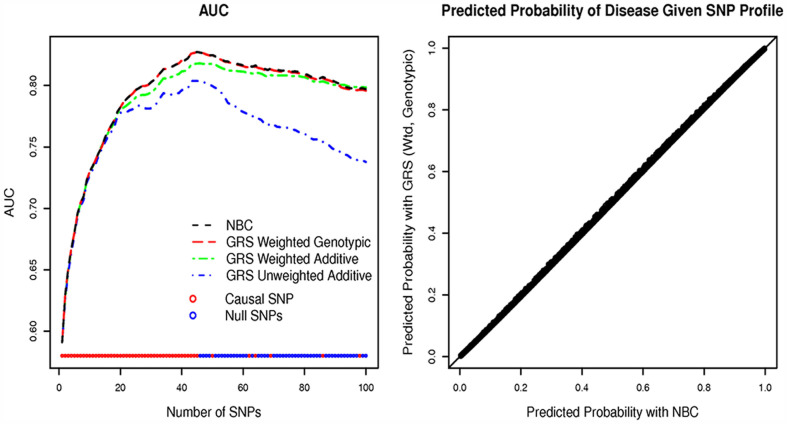
**Results of simulation for replication set**. The left hand plot graphs the mean area under the ROC (AUC) versus the number of SNPs in the prediction model. The colored lines refer to the AUC of the NBC (black), the unweighted GRS from an additive model (case 1, blue), the weighted GRS from an additive model (case 2, green), and the weighted GRS from a genotypic model (case 3, red). The maximum AUC occurs at 45 SNPs. The right hand plot graphs the probability of the trait *T* given the weighted GRS (genotypic model) on the *y*-axis versus the probability of disease given the SNP profile estimated by NBC on the *x*-axis for a model containing 45 SNPs for one of the replicates. The right hand plot is similar across the five replicates.

Figure 3 (left panel) shows the mean AUC across five replicates for the NBCs and logistic regression models for different GRSs, with increasing number of SNPs. As expected based on our mathematical calculations, the AUCs of the genetic risk models based on the NBCs and the GRSs with a genotypic weights are identical (Figure 3, left panel), and the predicted probabilities are almost identical (Figure 3, right panel). The weighted and unweighted GRS using an additive mode of inheritance have lower AUCs demonstrating the loss of accuracy with assuming additivity when some of the SNPs do not follow an additive mode of inheritance. Of course if all SNPs do in fact follow an additive model of the inheritance, the genotypic and additive prediction models would perform similarly. The trend of the AUC shows that accuracy keeps increasing as true positive SNPs are included in the model, and then declines when each classification model starts including false positive SNPs. The decline is more evident for the case 1 GRS, while both weighted GRS based on additive or genotypic associations appear to be more robust.

## Discussion

One of the selling points of genome-wide association studies was to discover genetic variants that are associated with increased susceptibility for disease and could be used for personalized diagnosis and prognosis. Initial results published for example in Meigs et al. (2008) and Paynter et al. (2010) however showed that genetic data added limited predicted values to well established risk factors of Type II diabetes and cardiovascular disease. These initial studies limited the attention to those SNPs that reached genome-wide significance and their effect was summarized into a GRS. Since then, a growing body of literature has shown the increased value of deeper mining of genome-wide association studies but inclusion of large number of SNPs in genetic risk model has continued to resort on GRSs (Cui, 2009; Goddard et al., 2009; Kooperberg et al., 2009; Purcell et al., 2009; Yang et al., 2010; Chen et al., 2011; Chibnik et al., 2011), while machine learning type methods continue to be rare regardless of some successful applications (Wei et al., 2009; Okser et al., 2010; Kang et al., 2011b; Sebastiani et al., 2012).

Our study shows that risk prediction based on a GRS is mathematically equivalent to risk prediction based on a NBC, when the same SNPs with the same mode of inheritance are used in the models. The equivalence is based on the fact that both models essentially base the prediction on a weighted average of ORs of the individual SNPs. While this equivalence establishes the validity of methods based on the NBC for genetic risk prediction and we hope will contribute to make this approach more popular in this field, it also shows that contrary to what stated in Okser et al. (2010) a NBC does not include interactions of SNPs but only additive genetic effects. However, the directed graphical model underlying a NBC can be extended to more general structures to include interactions between genes and/or environmental risk factors by maintaining the computations scalable to genome-wide genotype data and even whole genome sequence data (Sebastiani and Perls, 2008).

**Figure 4:**
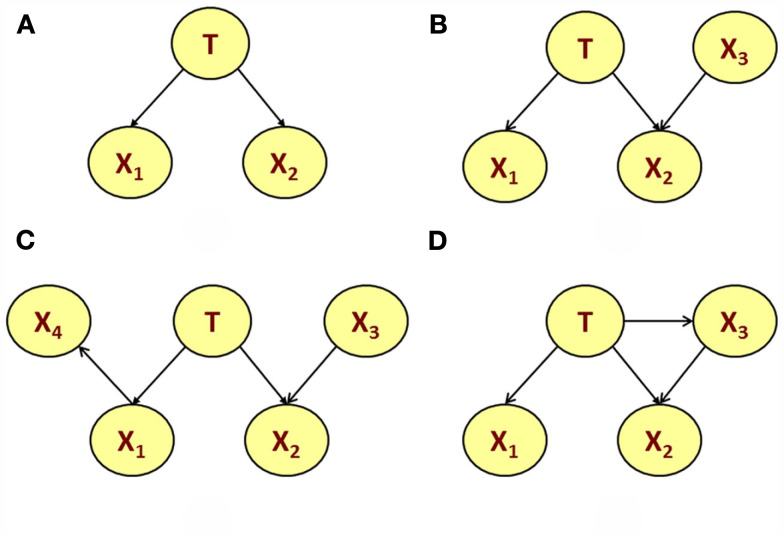
**Examples of directed acyclic graph (DAG)**. All nodes are random variables and the DAG represents Markov properties of marginal and conditional independence (Lauritzen and Sheehan, 2004). In particular, the global Markov property states that a node is independent of all other nodes in the DAG given its parent nodes, its children nodes and additional parents of its children (Lauritzen and Sheehan, 2004). In addition, two nodes are marginally independent when they have no directed joining paths after their children are dropped. Therefore, the nodes *X*_1_ and *X*_2_ in the DAG in **(A)** are conditionally independent given *T*. The DAG in **(B)** adds the node *X*_3_ to the NBC in **(A)**. This additional node is marginally independent of *T* but conditionally dependent on *T* given *X*_2_. The DAG in **(C)** includes an additional node *X*_4_ that is conditionally independent of all other nodes given *X*_1_. Finally, the DAG in **(D)** extends the DAG in **(B)** by adding a link from *T* to *X*_3_ so that *X*_3_ and *T* are marginally dependent.

Figure 4 shows some ways to extend NBCs for risk prediction to include population ancestry, as well as genetic and non-genetic effects that may be missed by test for marginal associations. Figure 4A describes a directed acyclic graph (DAG) with one parent node (*T*) and two children nodes (*X*_1_ and *X*_2_) that may represent SNPs. The DAG describes the conditional independence of *X*_1_ and *X*_2_ given *T*. This type of DAG with one root node and multiple conditionally independent children represents a NBC (Sebastiani and Abad-Grau, 2007). The DAG in Figure 4B extends the NBC in Figure 4A with an additional node *X*_3_ that is marginally independent of *T*, but conditionally dependent on *T* given *X*_2_. In the context of genetic risk modeling, the node *X*_3_ could represent a non-genetic risk factor that is associated with a trait *T* only in specific genetic backgrounds (the node *X*_2_). The DAG in Figure 4C includes an additional node *X*_4_ that is conditionally independent of all other nodes given *X*_1_. This additional node may represent a gene × gene interaction that is induced by linkage disequilibrium. Note that both DAGS in Figures 4B,C would give the same classification score for *T*, because of the independence of *T* from *X*_4_ given *X*_1_. So, the DAG in Figure 4C would be useful for a better explanation of the biology rather than improving genetic risk prediction. Finally, the DAG in Figure 4D extends the DAG in Figure 4B by adding a link from *T* to *X*_3_. The inclusion of this link makes the node *X*_3_ marginally dependent of *T* and interaction between *X*_2_ and *X*_3_ changes the classification score compared to the DAG in Figure 4B.

In addition, and most importantly, the fact that all variables in a DAG are random provides a sound framework for marginal and conditional inference. For example, a genetic risk model based on a DAG can be used for predicting the outcome of a subject by marginalizing out unobserved variables (Solovieff et al., 2011).

Our analysis is limited to binary outcomes, but we expect that similar results hold when the outcome to be predicted is a quantitative trait that follows a normal distribution. Furthermore, our analysis shows that linear transformations of a GRS do not impact predictive accuracy, and similarly, that the predictive accuracy of a NBC cannot be changed by a choice of prior for *T*. Improving the accuracy can be accomplished by selection of the most predictive SNP and by choosing alternative weights to calculate the GRS. There is no obvious similar choice for a NBC. However, a closely related approach that we used in Sebastiani et al. (2012) to improve the predictive accuracy is to use ensemble of nested NBCs. Finally, the machine learning community has developed many feature selection algorithms for building classifiers (Hastie et al., 2009) that, by the equivalence proved in this paper, may prove to be useful to generate better genetic risk models.

## Conflict of Interest Statement

The authors declare that the research was conducted in the absence of any commercial or financial relationships that could be construed as a potential conflict of interest.

## Appendix

### Derivation of property 3

$$\begin{array}{l} \sum_{i=1}^{k}\log\frac{p(T|S_{i})/(1-p(T|S_{i})}{p(T|S_{i}=\text{AA})/(1-p(T|S_{i}=\text{AA}))}>\frac{\tau -\gamma_{0}}{\gamma_{1}}\Rightarrow \text{classify as }T\\ \text{if and only if}\\ \sum_{i=1}^{\text{k}}\log\left(\frac{p(T|S_{i})}{1-p(T|S_{i})}\right)-\sum_{i=1}^{k}\log\left(\frac{p(T|S_{i}=\text{AA}}{1-p(T|S_{i}=\text{AA}}\right)>\frac{\tau -\gamma_{0}}{\gamma_{1}}\Rightarrow \text{classify as }T\\ \text{if and only if}\\ \sum_{i=1}^{k}\log\left(\frac{p(T|S_{i})}{1-p(T|S_{i})}\right)>\frac{\tau -\gamma_{0}}{\gamma_{1}}+\sum_{i=0}^{k}\log\left(\frac{p(T|S_{i}=\text{AA})}{1-p(T|S_{i}=\text{AA})}\right)\Rightarrow \text{classify as }T\\ \text{if and only if}\\ \sum_{i=1}^{k}\log\left(\frac{p(T|S_{i})}{1-p(T|S_{i})}\right)>\log(\lambda )\\ \text{where }\log(\lambda )=\frac{\tau -\gamma_{0}}{\gamma_{1}}+\sum_{i=1}^{k}\log\left(\frac{p(T|S_{i}=\text{AA})}{1-p(T|S_{i}=\text{AA})}\right)\\ \text{and }\tau =\gamma_{0}-\gamma_{1}\sum_{i=1}^{k}\log\left(\frac{p(T|S_{i}=\text{AA})}{1-p(T|S_{i}=\text{AA})}\right)+\gamma_{1}\log(\gamma ) \end{array}$$
